# Modular Nanotransporters Delivering Biologically Active Molecules to the Surface of Mitochondria

**DOI:** 10.3390/pharmaceutics15122687

**Published:** 2023-11-27

**Authors:** Yuri V. Khramtsov, Alexey V. Ulasov, Tatiana A. Slastnikova, Andrey A. Rosenkranz, Tatiana N. Lupanova, Georgii P. Georgiev, Alexander S. Sobolev

**Affiliations:** 1 Laboratory of Molecular Genetics of Intracellular Transport, Institute of Gene Biology of Russian Academy of Sciences, 34/5 Vavilov St., 119334 Moscow, Russia; ykhram2000@mail.ru (Y.V.K.); al.ulasov@gmail.com (A.V.U.); slacya@gmail.com (T.A.S.); aar@genebiology.ru (A.A.R.); tatyanalupanova@gmail.com (T.N.L.); georgiev@genebiology.ru (G.P.G.); 2 Faculty of Biology, Lomonosov Moscow State University, 1-12 Leninskie Gory St., 119234 Moscow, Russia

**Keywords:** modular nanotransporters, Keap1, intracellular delivery, CETSA, FLIM-FRET, FLIM, thermophoresis, chlorin *e*_6_, targeting to mitochondria

## Abstract

Treatment of various diseases, in particular cancer, usually requires the targeting of biologically active molecules at a selected subcellular compartment. We modified our previously developed modular nanotransporters (MNTs) for targeting mitochondria. The new MNTs are capable of binding to the protein predominantly localized on the outer mitochondrial membrane, Keap1. These MNTs possessing antiKeap1 monobody co-localize with mitochondria upon addition to the cells. They efficiently interact with Keap1 both in solution and within living cells. A conjugate of the MNT with a photosensitizer, chlorin *e*_6_, demonstrated significantly higher photocytotoxicity than chlorin *e*_6_ alone. We assume that MNTs of this kind can improve efficiency of therapeutic photosensitizers and radionuclides emitting short-range particles.

## 1. Introduction

Protein–protein interactions have been the focus of substantial scientific efforts over the past decades [1,2,3]. These efforts have resulted in the identification and evaluation of many proteins, including intracellular ones, as potential therapeutic targets [4,5]. Current strategies to modulate intracellular proteins encompass mainly two modalities: macromolecules and small molecules [6,7]. Although small-molecule inhibitors are highly amenable for some targets, practically speaking, they are mostly restricted to targets with suitable binding pockets, such as enzymes, receptors, and ion channels, leaving a large fraction of cellular proteins out of the scope of their regulation. The specificity problem and the unknown mechanism of some small molecules also limit their applicability [8,9]. Macromolecules, for instance, antibodies or antibody-like scaffolds, have addressed some of these concerns, as they could bind large areas on the protein surfaces, undruggable by small molecules. In fact, antibody therapeutics became the standard of care for different diseases, and nearly one hundred and fifty antibodies have been approved for clinical use [10,11]. Remarkably, the majority of clinically used antibodies are limited to transmembrane and secreted protein targets [12,13], excluding intracellular targets, whose overall number is estimated to be two-thirds of the whole cellular proteome [14]. Thereby, the delivery issue has mostly constrained the development of antibody-like molecules as mighty binding tools for intracellular proteins in living cells.

To explore this issue, a spectrum of approaches has been proposed. Transfection-based methods are the mainstay of intracellular antibody delivery [15]. However, efficient and safe transfection technologies are yet to be developed, sparking interest in alternative delivery opportunities, such as fusion with cell penetrating peptides, different types of nanocarriers, lipids, and polymers [16,17,18,19]. In this spirit, we have expanded our modular nanotransporters (MNT) platform to deliver intracellular protein binder inside cells [20]. MNT is a technological platform that is designed for targeted intracellular delivery of cytotoxic agents, such as photosensitizers, therapeutic radionuclides, and regulatory proteins [20,21,22]. Moreover, MNTs themselves do not have noticeable toxicity in the concentration ranges used [20,21]. MNTs impart cell specificity to a delivering cargo due to its modules providing “recognition” of the desired target cells and receptor-mediated endocytosis with the subsequent targeted intracellular transport into the desired cellular compartment. An endosomal escape (a necessary step to prevent MNT with cargo from degradation in lysosomes) is mediated via the MNT endosomolytic module. To date, we have successfully implemented MNT-mediated delivery of locally acting agents into the cell nucleus on various animal models, including experimentally induced melanoma, human epidermoid carcinoma, bladder and cervical cancers (reviewed in [20]). Mitochondria are another intracellular target which has attracted attention of many investigators dealing with development of anti-cancer medicines, especially because their damage causes apoptotic death of cancer cells [23,24,25].

As an intracellular target, we chose the Keap1 protein localized on the outer mitochondrial membrane, which is involved in the regulation of several cellular functions: normoxia maintenance, proteostasis, cytoskeleton regulation, and some others [26,27,28]. Its close proximity to the outer mitochondrial membrane may be exploited for the delivery of membrane-disrupting agents such as photosensitizers or radionuclides emitting particles with high linear energy transfer. In this article, we leveraged the benefits of cell-specific MNT technology to deliver regulatory proteins inside cells and tested the feasibility of this approach to transport the anti-Keap1 monobody to its target. We demonstrated efficient intracellular delivery of the anti-Keap1 monobody with several assays, such as fluorescence-lifetime imaging microscopy (FLIM), FLIM-FRET, and cellular thermal shift assay (CETSA). Finally, we confirmed that the photocytotoxicity of photosensitizer conjugated to the studied MNT was enhanced when compared to either free chlorin *e*_6_ or chlorin *e*_6_ delivered by control MNT lacking an anti-Keap1 monobody. This study is a part of our attempts to interfere with Keap1 functions and highlights the importance of protein delivery method development as a direct tool to regulate target proteins in the target cell. To investigate whether this phenomenon may be leveraged to develop anti-cancer drugs, cells expressing epidermal growth factor receptor (EGFR) were employed.

## 2. Materials and Methods

### 2.1. Cell Lines

The human epidermoid carcinoma A431 and murine hepatocyte AML-12 cell lines were obtained from the American Type Culture Collection (ATCC, Manassas, VA, USA) and maintained according to the ATCC specifications.

### 2.2. Modular Nanotransportsrs (MNT) Used in the Work

A DNA fragment coding for the antiKeap1 monobody was synthesized by General Biosystems (Morrisville, NC, USA) using already published amino acid sequence [29] and subcloned into the affibody(EGFR)-DTox-HMP-NLS plasmid [21] after NLS. The amino acid sequences of the affibody(anti-EGFR) [30] and the DTox-HMP-NLS [31] were published earlier. To obtain MNT_1_, NLS module was deleted using the QuickChange™ site-directed mutagenesis kit (Agilent Technologies, Santa Clara, CA, USA). The plasmids encoding MNT_1_ were verified by sequencing and retransformed in *E. coli* BL21(DE3) cells. The molecular weight of MNT_1_ is 83.3 kDa.

*E. coli* BL21(DE3) cells were grown on LB Broth Miller (Amresco, Solon, OH, USA) with ampicillin (100 μg/mL) to A600 = 0.8 at 37 °C. MNTs were expressed in *E. coli* by the addition of isopropyl-β-d-1-thiogalactopyranoside to a 0.5 mM final concentration at 18 °C overnight. The cell suspension was pelleted at 8900× *g* for 30 min at 4 °C using the JA-10 rotor (Beckman Coulter, Brea, CA, USA). Then the obtained pellets were lysed in ice-cold lysis buffer containing sodium phosphate (50 mM), sodium chloride (300 mM), pH 8.0, lysozyme (10 mg/mL), phenylmethylsulfonyl fluoride (1 mM), and Triton X-100 (0.5%). Following subsequent centrifugation (4 °C, 30 min, 28,000× *g*, JA-20 rotor) the clarified supernatant was loaded onto an Ni Sepharose 6 Fast Flow prepacked column (Cytiva, Marlborough, MA, USA). The column was then washed with washing buffer 1 containing sodium phosphate (50 mM), sodium chloride (300 mM), imidazole (20 mM), and glycerol (1%), Triton X-100 (0.5%), pH 8.0, followed by washing buffer 2 containing sodium phosphate (50 mM), sodium chloride (300 mM), and imidazole (20 mM), pH 8.0. Finally, the MNT was eluted from the column with elution buffer containing sodium phosphate (50 mM), sodium chloride (300 mM), and imidazole (700 mM), pH 8.0, and dialyzed against phosphate buffered saline (10 mM sodium phosphate, 150 mM sodium chloride, pH 7.4).

As a control MNT, we used MNT similar to MNT_1_ but lacking an antiKeap1 monobody fragment (MNT_0_). The molecular weight of MNT_0_ is 72.5 kDa. MNT_0_ and MNT_1_ concentrations were measured using the Bradford method.

### 2.3. Flow Cytometry

Flow cytometry was used to study the ability of MNT_1_ to bind to EGFR receptors on the surface of AML-12 cells. The MNT_1_ was labeled with the AF488 fluorescent dye in the following way: an 8-fold molar excess of the activated AF488-N-hydroxysuccinimide ester (Lumiprobe, Moscow, Russia) was mixed with MNT_1_ in carbonate buffer (65 mM NaHCO_3_, 83 mM NaCl, pH 8.5), and subsequently stirred constantly for 1 h at room temperature. The MNT_1_ with the attached AF488 was purified from unreacted dye using a PD10 chromatographic column (GE Healthcare, Buckinghamshire, UK). Using a molar extinction coefficient of 71,800 L·mol^−1^·cm^−1^ at a wavelength of 519 nm, the concentration of AF488 attached to MNT_1_ was calculated. MNT_1_ concentration was measured using the Bradford method. As a result, an average of 3.3 AF488 molecules are attached to one MNT_1_ molecule. AML-12 cells seeded in a 24-well plate were incubated with 500 nM MNT_1_ for a given time, washed twice with Versene solution, then removed with a 0.25% trypsin solution in Versene, and transferred to Hank’s solution. The amount of MNT_1_-AF488 bound to cells was determined using a CytoFLEX S flow cytometer (Beckman Coulter, Inc., USA) in the fluorescence channel of 500–550 nm. Fluorescence was excited by a laser with a wavelength of 488 nm. The average fluorescence value per cell was determined from 7–13 replicates at different incubation times with MNT_1_-AF488. Large cell aggregates were excluded from consideration.

### 2.4. Liposome Leakage Assay

The ability of MNT_1_ to provide liposome leakage was demonstrated on unilamellar phosphatidylcholine (Sigma-Aldrich, Burlington, MA, USA) liposomes loaded with the fluorescent dye calcein (Fluka, Seelze, Germany). Unilamellar liposomes were loaded with fluorescent dye calcein to the concentration of 100 mM which is self-quenching. To do this fresh lipid suspension in liposome buffer containing HEPES (20 mM), MES (20 mM), citrate (20 mM), and sodium chloride (150 mM), pH 7.4 was sonicated until clear, using a W-181-T sonicator (Ulta Sonic Finland LTD, Lanti, Finland; 40 kHz, 90 W, 0 °C, 30 min), Then the resulting liposomes were passed 10 times through 0.22 µm Durapore filters (Millipore, Burlington, MA, USA) for size standardization. The liposomes were stored at 4 °C under an argon atmosphere. Liposomes were purified on PD-10 prior to experiment, then the same day incubated for 30 min with 100 nM MNT_1_ in liposome buffer at the different pHs at room temperature in triplicate. After that, samples were diluted tenfold in liposome buffer pH 7.5, and the fluorescence of free calcein (leaked from liposomes) was measured at 520 nm (excitation at 490 nm). As a positive control (100% calcein leakage), a 0.5% Triton X-100-containing sample was used. Parallel probes where MNT_1_ was omitted served to assess background calcein leakage.

### 2.5. Thermophoresis

MNTs and Keap1 interaction was assessed using a Monolith NT.115 instrument (NanoTemper Technologies, Munchen, Germany) in phosphate buffer containing sodium phosphate (25 mM) and sodium chloride (150 mM), pH 8.0. Keap1 was labeled with Cy3 fluorescent dye. Then, 10-fold molar excess of Cy3 hydroxysuccinimide ester (Lumiprobe, Moscow, Russia) was mixed with Keap1 in 65 mM carbonate buffer (pH 8.5), followed by 1 h incubation at room temperature, stirring constantly. Keap1 with attached Cy3 was purified from unreacted Cy3 via a PD10 column. As a result, a modification ratio of 3.9 Cy3 molecules per one Keap1 molecule was attained. Thermophoretic curves were obtained at a fixed concentration of Keap1-Cy3 (40 nM). The obtained dissociation constants for the MNT complex with Keap1, K_d_, were averaged over 8–16 curves.

### 2.6. FLIM-FRET

To study the interaction of MNT_1_ with Keap1 in the target cell, MNT_1_ was labeled with the N-hydroxysuccinimide ester of the fluorescent dye AF568 (Lumiprobe) by incubation with a 4-fold molar excess of the fluorophore in sodium carbonate buffer, pH 8.6, for 1 h at room temperature. Purification of labeled MNT_1_ with simultaneous buffer exchange to PBS, pH 7.5, was performed by gel filtration on columns with Sephadex G-25 (PD-10). The degree of modification of MNT_1_ by the fluorophore, estimated spectrophotometrically, was 1.3 AF568 molecules per one MNT_1_ molecule.

The interaction of MNT_1_ with Keap1 in the cell was demonstrated using fluorescence lifetime-based fluorescence resonance energy transfer (FLIM-FRET) [32,33]. The AML-12 cells transiently transfected with Keap1 protein fused to the fluorescent protein hrGFP were used as target cells (hrGFP-Keap1, Addgene plasmid # 28025). The cells were seeded into Poc-mini microscopy chambers at a cell density of 100 thousand per chamber in 1 mL of DMEM supplemented with 10% fetal bovine serum. hrGFP-Keap1 was transfected into AML12 cells according to the instructions of K2^®^ Transfection System (Biontex Laboratories GmbH, Martinsried, Germany). Two days later, the medium was changed to serum-free medium with 0.2% BSA, and AF568-labeled MNT_1_ was added to a final concentration of 500 nM, followed by incubation for 15 min or 1 h. At the end of the incubation, the medium was changed to the Hank’s solution with 0.2% BSA. In the control, no MNT_1_ was added to the cells.

FLIM-FRET experiments were performed on a multiphoton confocal laser scanning microscope LSM-510 META NLO (Carl Zeiss, Oberkochen, Germany), equipped with a Mai Tai Broadband femtosecond laser (SpectraPhysics, Irvine, CA, USA) and a time-correlated single-photon counting system (TCSPC from Becker & Hickl GmbH, Berlin, Germany) with a 63× lens, NA 1.4. hrGFP fluorescence was excited with a femtosecond laser using two-photon excitation at 800 nm. Fluorescence recording was carried out at wavelengths of 500–550 nm for hrGFP.

### 2.7. Cellular Thermal Shift Assay (CETSA)

The cellular thermal shift assay (CETSA) [34,35,36,37,38] was performed in the following way. Cells from 25 cm^2^ cultural flask were trypsinized and pelleted (200× *g*, 5 min). The obtained cell pellets were resuspended in buffer containing sodium phosphate (25 mM), sodium chloride (150 mM), EDTA (5 mM), phenylmethylsulfonyl fluoride (0.174 mg/mL), and aprotinin (1.5 μg/mL), pH 8.0. Cells were counted with the help of a MACSQuant Analyzer flow cytometer (Miltenyi Biotec GmbH, Paris, France). Both cell lysates (lysed by four freeze–thaw cycles) and intact cells were used to obtain melting curves of the evaluated proteins.

At the first step, cell lysates and intact cells were heated for 3 min to the pre-determined temperatures within the 40–50 °C range. After subsequent cooling down to room temperature, intact cells were lysed by four liquid nitrogen freeze–thaw cycles.

Then all cell lysates were spun down (8000× *g*, 4 °C, 60 min). The resulting supernatants were loaded onto 10% SDS-PAGE gel followed by a Western blot, stained for Keap-1 protein (anti-Keap1 antibody (ab139729, Abcam, Cambridge, UK)). In order to mitigate the effect of the frequent band inhomogeneous coloration detected on Western blot, each sample was loaded in triplicate. This enabled us to exclude random outliers of individual bands from the analysis, thus leading to more reliable averaged data. The Trans-Blot Turbo Transfer System (Bio-RAD, Hercules, CA, USA) was used to transfer the proteins from the gel to a 0.22 μm supported nitrocellulose membrane. The band signal intensity in the selected area as well as the background area intensity were measured, followed by background subtraction. Thus, the measured band intensity was averaged over three replicates loaded on gel and normalized to the band intensity of the sample, in which the heating step for CETSA was omitted. The resulting curve was fitted by the logistic sigmoid function using the Origin 6.0 software. Each experiment was performed in *n* = 3–9 replicates.

### 2.8. Labeling of Epidermal Growth Factor and MNT with ^125^I

1,3,4,6-tetrachloro-3α,6α-diphenylglycoluril, Iodogen (Sigma-Aldrich, Burlington, MA, USA) was used to label human epidermal growth factor (EGF, Sigma Chemicals, St. Louis, MO, USA) with ^125^I (Khlopin Radium Institute, Saint Petersburg, Russia). For this purpose, 10 µg of the EGF and 20 MBq of ^125^I sodium iodide in 0.05 M sodium borate buffer (pH 8.5) were incubated for 15 min at room temperature in glass vials coated with 10 µg of Iodogen. The resulting labeled EGF was purified through a PD-10 gel filtration column in phosphate-buffered saline (pH 7.5). Labeling and purification of MNT_1_ was accomplished in a similar manner.

### 2.9. Binding and Internalization Studies

The affinity of MNT_1_ to EGFR was estimated by binding ^125^I-iodoEGF to EGFR-expressing A431 cells. To examine the competitive binding of EGF and MNT_1_ to cells, 1 nM of ^125^I-iodoEGF and various concentrations of MNT_1_ were incubated overnight at 4 °C in 48-well plates in 200 µL of sodium carbonate-free DMEM with 20 mM 4-(2-hydroxyethyl)-1-piperazine-ethanesulfonic acid (HEPES, Sigma-Aldrich, Burlington, MA, USA) and 1% bovine serum albumin (BSA), pH 7.5. After the incubation, wells were quickly washed with ice-cold Hank’s solution four times and lysed for 30 min in 1 M NaOH. The cell lysates radioactivity was measured using a RiaGamma 1271 counter (LKB Wallac, Turku, Finland). The dissociation constants (K_d_) of the MNT_1_-receptor complexes were calculated according to the competitive model of the binding of ligands to receptors as described earlier [21]. The experiments were performed in triplicate, with error bars on graphs representing the standard error of mean (SEM).

A study of the internalization of ^125^I-iodo MNT_1_ was carried out on A431 cells. Cells were grown in DMEM with 10% FBS and gentamicin (50 μg/mL) at 37 °C and 5% CO_2_. Cells were seeded into 24-well culture plates, 500 thousand cells per well. Two days later, the medium was changed to DMEM without bicarbonate, supplemented with 10 mg/mL bovine serum albumin and 15 mM HEPES, pH 7.3. Radiolabeled MNT_1_ was added to the wells to a final concentration of 50 nM and incubated at 37 °C. At the end of incubation, the cells were washed on ice 4 times with a cold Hank’s solution, incubated at 4 °C for 1 h with a 0.25% trypsin in Versene solution to remove bound MNT_1_ from the cell surface, and washed additionally with the same solution. Under these conditions, 90% of the radioactivity associated with the cell surface became detached while the cell remained attached to the well. Experiments were carried out 3–4 times for each.

### 2.10. Intracellular Localization

To study localization in the target cell, MNT_1_ was labeled with the N-hydroxysuccinimide ester of the fluorescent dye AF488 (Lumiprobe, Moscow, Russia) by incubation with an 8-fold molar excess of the fluorophore in sodium carbonate buffer, pH 8.6, for 2.5 h at room temperature. Purification of labeled MNT_1_ with simultaneous buffer exchange to PBS, pH 7.5, was performed by gel filtration on PD-10 columns. The degree of modification of MNT_1_ by the fluorophore, estimated spectrophotometrically, was 2.3 AF488 residues per MNT_1_ molecule.

A431 cells expressing EGFR were used as target cells to study the intracellular localization of MNT_1_. The cells were seeded into Poc-mini microscopy chambers at a cell density of 100 thousand per chamber in 1 mL of DMEM with 10% FBS. Two days after, the medium was changed to serum-free medium with 0.2% BSA, and AF488 labeled MNT_1_ was added to a final concentration of 500 nM, followed by 1 h incubation. Fifteen minutes before the end of incubation, the mitochondria-specific dye MitoTracker Deep Red FM (Invitrogen, Carlsbad, CA, USA) was added to the chambers at a final concentration of 100 nM to visualize these organelles. At the end of the incubation, the medium was changed to the Hank’s solution with 0.2% BSA.

The intracellular distribution of MNT_1_ was studied using fluorescence lifetime imaging microscopy (FLIM) [32,33]. The advantage of this approach is that in a given area of the sample, not only the magnitude of the fluorescent signal in the selected spectral range is recorded, but also the fluorescence lifetime, which makes it possible to separate the signal from dyes with similar spectral properties. Experiments were performed on a multiphoton confocal laser scanning microscope LSM-510 META NLO (Carl Zeiss, Oberkochen, Germany), equipped with a Mai Tai Broadband femtosecond laser (SpectraPhysics, Irvine, CA, USA) and a time-correlated single-photon counting system (TCSPC from Becker & Hickl GmbH, Berlin, Germany) with a 63× lens, NA 1.4. AF488 fluorescence was excited with a femtosecond laser using two-photon excitation at 800 nm, and MitoTracker RED FM fluorescence was excited at 890 nm. Fluorescence recording was carried out at wavelengths of 500–550 nm and 575–640 nm for MNT_1_-AF488 and MitoTracker RED FM, respectively.

### 2.11. Photocytotoxicity

Photosensitizer chlorin *e*_6_ was obtained from Frontier Scientific, Philadelphia, PA, USA. Chlorin was coupled to MNT using 1-ethyl-3-(3-dimethylaminopropyl)-carbodiimide and N-hydroxysuccinimide (Sigma-Aldrich, Burlington, MA, USA) A 3 mM aqueous solution of chlorin *e*_6_ was diluted in activation buffer containing 10 mM MES (Sigma-Aldrich, Burlington, MA, USA) pH 6.5, and 1 mM sodium dodecyl sulphate (SDS-Na) to a final concentration of 150 μM. The resulting solution was mixed with freshly prepared aqueous solutions of the bifunctional cross-linking agent 1-ethyl-3-(3-dimethylaminopropyl)-carbodiimide (0.1 M; Sigma, USA) and N-hydroxysuccinimide (0.1 M; Sigma-Aldrich, Burlington, MA, USA) in the molar ratio PS: carbodiimide:N-hydroxysuccinimide = 1:4.5:15). The reaction mixture was thoroughly mixed and incubated for 1 h at a temperature of 25 °C with constant stirring. Then this activated chlorin *e*_6_ solution was added gradually (in portions of 10% of the total volume of the solution with an interval between portions of at least 5 min, temperature 25 °C) to 10 μM solution of MNT in buffer containing 30 mM sodium phosphate, 20 mM sodium borate, 150 mM sodium chloride, pH 8.0, and SDS-Na in the molar ratio MNT:SDS-Na = 1:5. Then, the reaction mixture was incubated on ice for 19 h with constant stirring. The reaction was stopped by the addition of hydroxylamine (Reakhim, Moscow, Russia) to a final concentration of 10 mM, after which the conjugate was incubated at 4 °C for 1 h with constant stirring. Conjugates were purified by affinity chromatography on Ni-NTA Sepharose (Cytiva, Marlborough, MA, USA). The A431 cells were seeded into 96-well plates (1000 cells/well). The next day, the medium was changed to DMEM with 10 mg/mL bovine serum albumin, and various concentrations of MNT-chlorin *e*_6_ or free chlorin *e*_6_ were added to the cells. After 20 h incubation, cells were washed two times with Hank’s solution and illuminated for 10 min (irradiation power 0.0276 W/cm^2^) with a slide projector (100% cell survival without free chlorin *e*_6_ or MNT-chlorin *e*_6_) in Hank’s solution. Then Hank’s solution was replaced with fresh media supplemented with 10% fetal bovine serum and grown for 4–5 days. Cell viability was determined using 3-(4,5-dimethylthiazol-2-yl)-2,5-diphenyltetrazolium bromide (MTT).

## 3. Results

**Figure 1:**
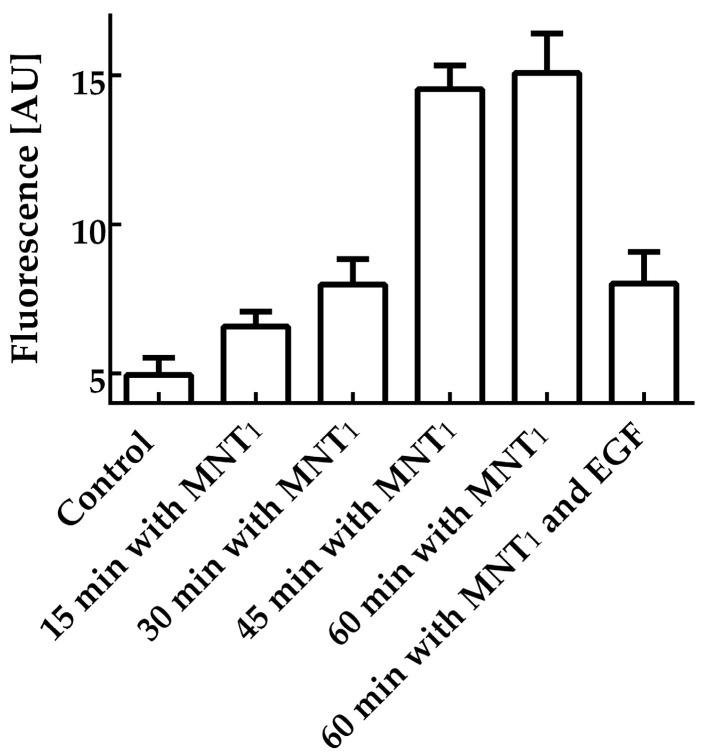
Interaction of MNT_1_ fluorescently labeled with AF488 with AML-12 cells possessing EGFR (arbitrary units). A control group, where MNT_1_-AF488 was omitted, groups in which cells were incubated with 500 nM MNT_1_-AF488 for 15, 30, 45, and 60 min, and a group in which cells were incubated with 500 nM MNT_1_-AF488 in the presence of 2 μM EGF are shown. Bars represent mean values ± SEM (*n* = 9–18).

Flow cytometry was used to study the interaction of MNT_1_ with EGFR-expressing cells. EGFR-positive AML-12 cells were incubated for various times with 500 nM MNT_1_ labeled with the fluorescent dye AF488. According to this study, just 15-min incubation of MNT_1_-AF488 with cells increased the average fluorescence significantly (*p* < 0.0001, *t*-test) over the fluorescence of control cells, where MNT_1_ was omitted (Figure 1). MNT_1_ possesses an affibody for the EGFR. To study whether the MNT_1_ interaction is EGFR-specific, a control where MNT_1_-AF488 was incubated for one hour with an excess of EGF (2 μM) was also measured. The presence of an excess of EGF led to significant (*p* < 0.0001, *t*-test) reduction of the average fluorescence compared to the cells incubated with MNT_1_-AF488 alone (Figure 1). Thus, affibody in MNT_1_ retains its ability to interact with EGFR-positive cells in an EGFR-specific manner.

**Figure 2:**
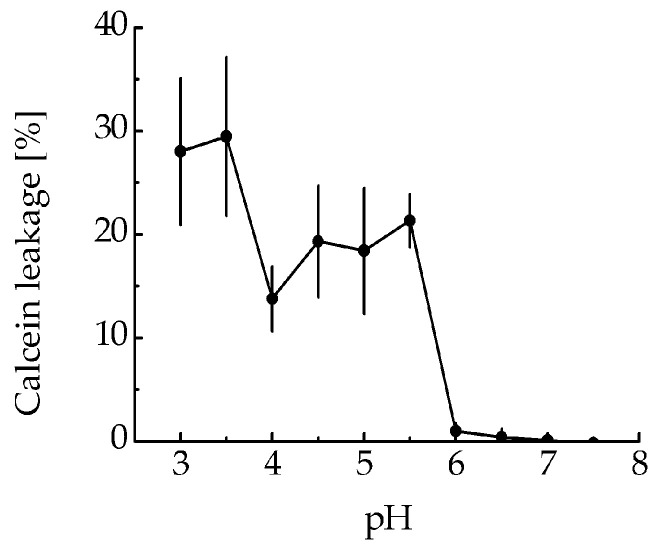
MNT_1_-induced leakage from phosphatidylcholine liposomes loaded with the fluorescent dye calcein to the fluorescence self-quenching concentration. MNT_1_ concentration is 100 nM. Error bars represent SEM (*n* = 6).

The ability of MNT_1_ to make membrane pores was tested, assessing calcein leakage from model phosphatidylcholine liposomes at various pHs. MNT_1_ did not possess any liposome disturbing activity at neutral pH (Figure 2), but led to significant liposome leakage in a slightly acidic medium (pH 5–5.5).

As we showed earlier using the thermophoresis method in solution, the equilibrium dissociation constant of the MNT_1_ complex with Keap1, K_d_, is equal to 7.9 ± 3.3 nM [38]. Thus, the preservation of the functional activity of the monobody module within the MNT_1_ molecule has already been confirmed. Using thermophoresis, we found that for the complex of MNT_0_ with Keap1, K_d_ = 93 ± 15 nM (Figure S1a). It was also shown that both the free affibody (Figure S1b) and the free translocation domain of diphtheria toxin, DTox (Figure S1c), bind to Keap1 with K_d_ > 1 µM, i.e., for both of them only low-affinity nonspecific binding is observed. On the contrary, for an MNT consisting of two modules—DTox and a carrier module, HMP—K_d_ is 93 ± 11 nM (Figure S1d). Thus, in addition to monobody, the HMP carrier module can also interact with Keap1.

**Figure 3:**
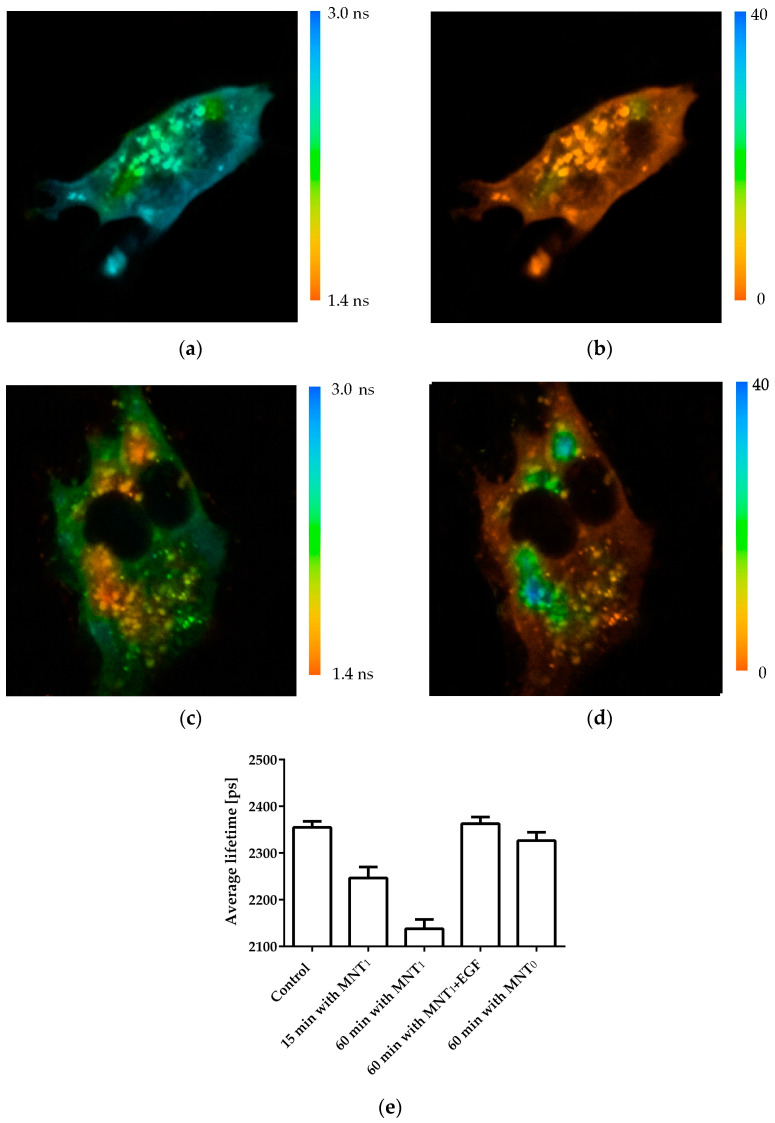
Analysis of the interaction of intracellular Keap1-hrGFP with MNT_1_-AF568 added to AML-12 cells expressing hrGFP-Keap1. Distribution of the average lifetime of hrGFP fluorescence in a cell to which no MNT was added (**a**) and in a cell following 1 h incubation with 500 nM MNT_1_-AF568 (**c**). Standard deviation, χ^2^, of the observed drop in hrGFP fluorescence intensity from a single-exponential dependence in a cell to which no MNT was added (**b**) and in a cell following 1 h incubation with 500 nM MNT_1_-AF568 (**d**). To display only the transformed hrGFP-Keap1 cell, a mask was applied to the image. (**e**) Average hrGFP fluorescence lifetime for different experimental groups of Keap1-hrGFP transformed AML-12 cells. Control group without the addition of MNT_1_, experimental groups where the cells were incubated with 500 nM MNT_1_-AF568 for 15 and 60 min, a group where the cells were incubated with 500 nM MNT_1_-AF568 in the presence of 2 μM EGF, and a group where cells were incubated with 500 nM MNT_0_-AF568 for 60 min. Data are presented as average hrGFP fluorescence lifetimes in a cell ± SEM (*n* = 9–18).

The interaction of MNT_1_ with Keap1 in the cell was demonstrated using fluorescence lifetime-based fluorescence resonance energy transfer (FLIM-FRET) [32,33]. To do this, AML-12 cells were transiently transfected with Keap1 protein fused to the green fluorescent protein hrGFP. MNT_1_ was labeled with the fluorescent dye AF568. In the case of FRET between these dyes, the fluorescence lifetime of the hrGFP donor should decrease [32,33]. In addition, in this case, a single exponential curve should no longer adequately fit the decrease in hrGFP fluorescence. In other words, the standard deviation, χ^2^, of the observed fluorescence drop from the single-exponential dependence should increase greatly. Incubation of AML-12 cells expressing Keap1-hrGFP with 500 nM MNT_1_-AF568 demonstrates both a decrease in the average hrGFP lifetime and a noticeable increase in χ^2^ of some areas of the studied cells (Figure 3). This indicates the presence of FRET between Keap1-hrGFP and MNT_1_-AF568 in these regions, and hence the interaction between Keap1 and MNT_1_. The average lifetime of hrGFP significantly decreases (*p* < 0.01, Dunnett test) after 15 min of incubation of AML-12 cells with MNT_1_-AF568. In contrast, incubation of AML-12 cells with 500 nM MNT_0_ for 1 h does not significantly reduce the fluorescence lifetime of Keap1-hrGFP (Figure 3e). When AML-12 cells expressing Keap1-hrGFP were incubated for 1 h with 500 nM MNT_1_-AF568 in the presence of 2 µM EGF, the average fluorescence lifetime of Keap1-hrGFP did not significantly differ from the fluorescence of cells to which MNT_1_ was not added (Figure 3e), indicating EGFR-dependent accumulation of the MNT_1_ in these cells. These data points indicate the ability of MNT_1_ to interact with Keap1 in the cytosol of EGFR-positive AML-12 cells.

**Figure 4:**
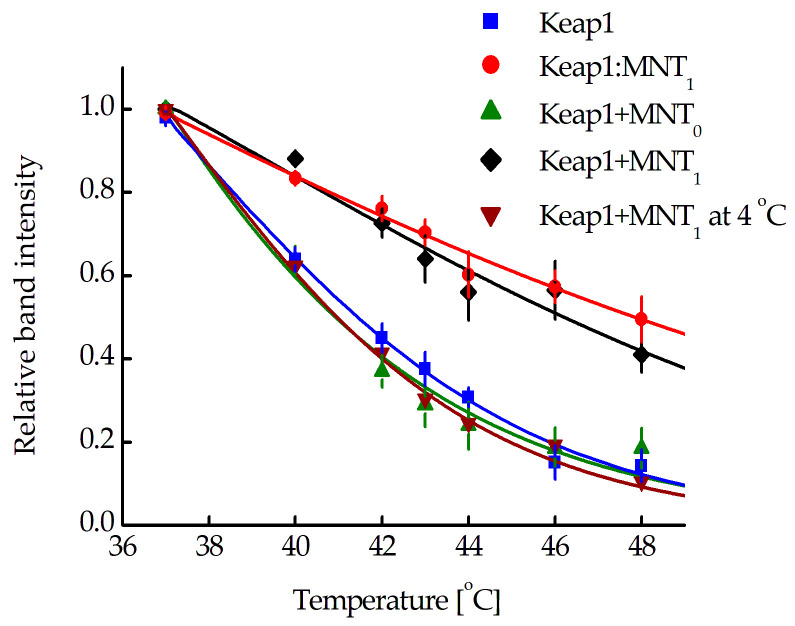
Melting curves of Keap1 in its natural protein environment (blue curve), Keap1 bound to MNT_1_ (red curve), Keap1 after 15 min incubation of cells with 500 nM MNT_1_ (black curve), Keap1 after 15 min incubation of cells with 500 nM MNT_0_ (green curve), and Keap1 after 15 min incubation of cells with 500 nM MNT_1_ at 4 °C (wine curve) in AML-12 cells. The dependences are normalized to the band intensity at 37 °C. Standard errors (SE) are shown (*n* = 3–9).

The interaction of MNT_1_ with Keap1 in AML-12 cells was also studied using cellular thermal shift assays (CETSA) [34,35,36,37,38]. Using this method, a Keap1 melting curve in its natural protein environment was obtained in AML-12 cells (Figure 4, blue curve). The curve corresponding to the Keap1 complex with MNT_1_ was obtained for the AML-12 cell lysate to which 1 µM MNT_1_ was added (Figure 4, red curve). The Keap1 melting curve obtained after 15-min incubation of AML-12 cells with 500 nM MNT_1_ followed by subsequent washing of cells (Figure 4, black curve), coincides with the melting curve for the Keap1-MNT_1_ complex. In contrast, when AML-12 cells were treated with 500 nM MNT_0_ for 15 min, the Keap1 melting curve (Figure 4, green curve) corresponded to the Keap1 melting curve obtained from cells incubated without MNT. The Keap1 melting curve obtained at 4 °C for AML-12 cells that were incubated for 15 min with 500 nM MNT_1_ matches the Keap1 melting curve of cells without MNT_1_ (Figure 4, wine curve). Thus, the CETSA method showed that after 15 min incubation with cells, MNT_1_ is able to interact with Keap1 in the cytosol, while MNT_0_ is not. Low incubation temperatures, which suppress the process of endocytosis, inhibited MNT_1_ binding to Keap1 in the cytosol.

**Figure 5:**
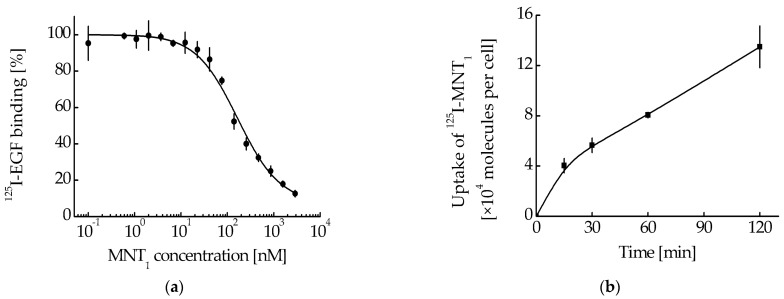
(**a**) Competition for binding to human epidermoid carcinoma A431 cells with ^125^I-EGF for MNT_1_ (% of control). Error bars are SEM (*n* = 3). (**b**) Uptake of ^125^I-MNT_1_ by A431 cells. ^125^I-MNT_1_ was added to cells seeded in 24-well plates to a final concentration of 50 nM in DMEM medium with 10 mg/mL BSA and incubated at 37 °C. At the indicated time intervals, cells were washed, treated with trypsin to remove surface-bound MNT_1_, and lysed by adding 1 M NaOH. Data are presented as means ± standard error (*n* = 3–4).

So, MNT_1_ is able to interact with the Keap1 protein, both in solution and in the cell. In turn, a noticeable proportion of Keap1 is located on the surface of mitochondria. To demonstrate the mitochondrial localization of MNT_1_, the cancer cell line A431 was used. This line also showed effective binding of MNT_1_ to EGFR. The dissociation constants (K_d_) for the MNT_1_ obtained from the displacement curve (competition of the unlabeled MNT_1_ with ^125^I-EGF, Figure 5a) was 63 nM (95% confidence interval, 51–76 nM). Moreover, MNT_1_ not only can bind to EGFR on the surface of A431, but also can be internalized into these cells. The uptake of ^125^I-labeled MNT_1_ by A431 cells is presented in Figure 5b. Thus, MNT_1_ enters A431 cells and can interact with Keap1 in them, which, in turn, can lead to mitochondrial localization of MNT_1_.

**Figure 6:**
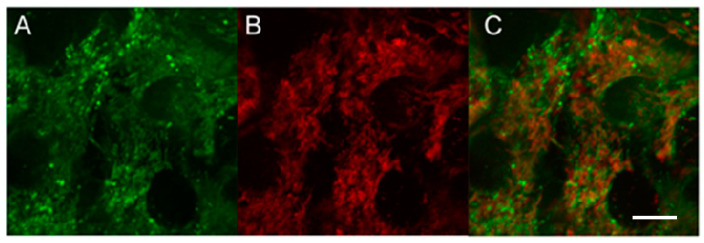
The intensity of the signal recorded in A431 cells in the fluorescence channels AF488-MNT_1_ (**A**) and Mito Tracker red FM (**B**) upon multiphoton excitation of fluorescence at 800 nm (**A**) and 890 nm (**B**) 1 h after incubation with AF488 labeled MNT_1_ and 15 min incubation with Mito Tracker red FM. (**C**) is an overlay of (**A**,**B**). Bar is 10 μm.

MNT_1_ intracellular localization studies were performed in EGFR-positive A431 cells. The presence of intracellular sites with a simultaneous signal from MNT_1_ and a mitochondria-specific dye (Figure 6), resulting in the appearance of orange-colored areas in the image, indicates the colocalization of dyes within the cell (Figure 6C).

**Figure 7:**
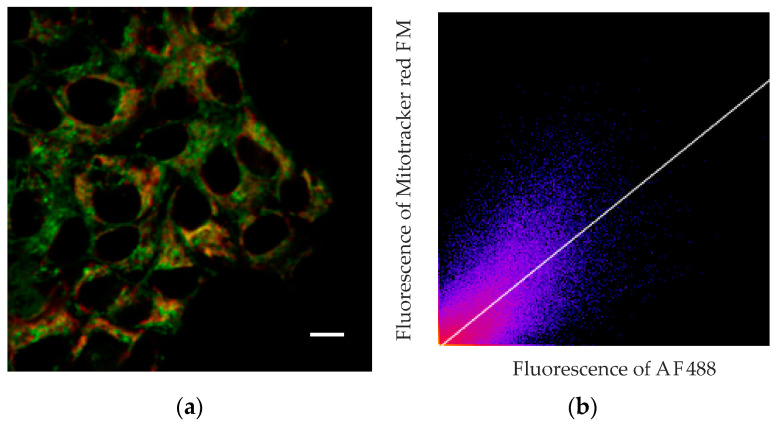
Detection of colocalization of fluorescence of AF488 labeled MNT_1_ and mitochondria. (**a**)—Image of the fluorescence intensity of the mitochondria-specific dye MitoTracker red FM (red) and MNT_1_ labeled with the dye AF488 (green). Bar is 10 μm. (**b**)—Graph of the correlation between the fluorescence intensity of the MitoTracker red FM and AF488 dyes presented in panel (**a**). The correlation coefficient of the regression line shown in the graph is 0.77.

To assess the extent of this colocalization, we used fluorescence-lifetime imaging microscopy (FLIM). Fluorescence decay studies showed that the fluorescence decay curves of the dyes fitted well to a single exponential relationship, and the fluorescence lifetimes for AF488 and MitoTracker RED FM differed greatly. MitoTracker RED FM in A431 cells shows an average fluorescence lifetime (µ) of 1203 ps (confidence interval, CI, was 1085–1381 ps), while the average lifetime of the AF488 dye attached to MNT averages 2140 ps (CI 1970–2315 ps). The lifetimes of the cell autofluorescence signal in the signal measurement channels from both AF488 and MitoTracker differed significantly in their lifetimes from the dyes and were 2473 ps (CI 2108–2899 ps) and 2710 ps (CI 2515–2802 ps), respectively. The magnitude of the autofluorescent signal was significantly inferior to the magnitude of the dye signal, even under optimal conditions for autofluorescence measurement, and amounted to no more than 20% of the AF488 signal and 2–3% of the MitoTracker RED FM signal. Such a difference in the signal intensity and the lifetimes of dyes and the background signal and separation of images by lifetimes makes it possible to almost completely eliminate the contribution of autofluorescence from the resulting images. Fluorescence colocalization analysis of AF488 and MitoTracker RED FM showed a strong correlation between the fluorescence from the mitochondria-specific dye and the fluorescence of the dye attached to MNT_1_ (Figure 7). The linear correlation coefficient (Pearson’s correlation coefficient) calculated from the obtained data using the built-in Coloc 2 plugin of the ImageJ image processing program showed a significant correlation (0.77, *p* < 0.001) between the fluorescence intensity of both dyes at their colocalization sites. Spearman’s rank correlation coefficient, showing the monotonicity of the dependence of the variables, was 0.87, which confirms the presence of a relationship between the fluorescence intensity of both dyes. The data obtained indicate colocalization of AF488-MNT_1_ fluorescence and the dye on mitochondria.

**Figure 8:**
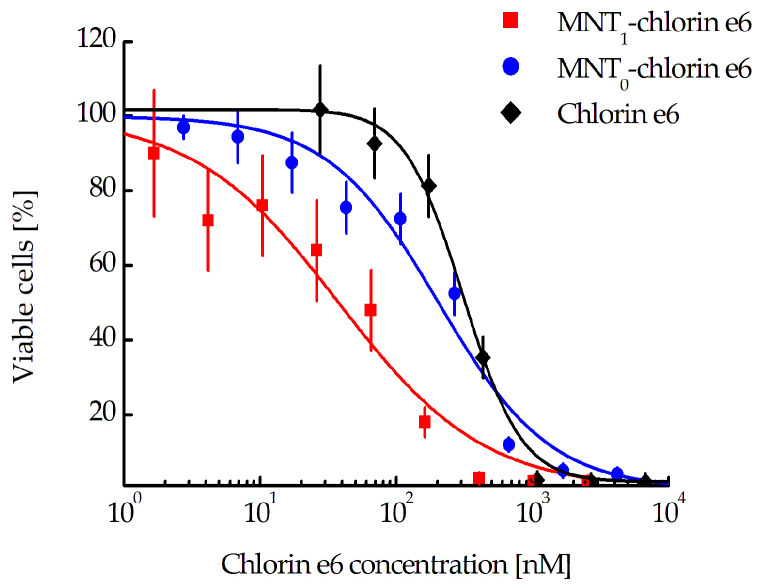
Photocytotoxicity of MNT_1_-chlorin *e*_6_ conjugate compared to either free chlorin *e*_6_ or MNT_0_-chlorin *e*_6_ on A431 cells. Error bars represent SEM (*n* = 6–10). The lines show the interpolation of the experimental data of the logistic sigmoid curves (four-parametric model).

Evaluation of the photocytotoxicity on EGFR-positive human epidermoid carcinoma A431 cells demonstrated significant enhancement (8.6 and 5.4 times, respectively) of the efficacy of chlorin *e*_6_ attached to MNT_1_ (EC_50_ = 37.7 nM, CI 23.3–59.4 nM) when compared to either free chlorin *e*_6_ (EC_50_ = 324.7 nM, CI 290.1–362.4 nM) or chlorin *e*_6_ attached to control MNT_0_ lacking an antiKeap1 monobody fragment (EC_50_ = 204.1 nM, CI 137.8–300.7 nM) (Figure 8).

## 4. Discussion

To deliver biologically active molecules to a selected compartment of target cells, we proposed the technology of modular nanotransporters (MNTs) [20]. MNTs can bind to an internalizable receptor on the surface of target cells through a ligand module. After internalization and entry into endosomes, due to the activity of the endosomolytic module within them, MNT can be released into the cytosol. Delivery to the selected compartment or target protein is carried out using an intracellular targeting module. The delivered molecule can be attached to a carrier module, which also links the other modules together. In this study, an antibody-like molecule, affibody, to the human EGFR [30,39] was chosen as a ligand module. *E. coli* hemoglobin-like protein (HMP) served as a carrier module, while the translocation domain of diphtheria toxin (DTox) was chosen as an endosomolytic module. We used the Keap1 protein, a significant portion of which is located on the surface of mitochondria [40], as a target protein. To bind to Keap1, another antibody mimetic, an anti-Keap1 monobody [29], was included within the MNT_1_ molecule. The complete transporter in this work was designated MNT_1_, and control MNT lacking the anti-Keap1 monobody was designated MNT_0_.

First of all, it was necessary to test whether each module within MNT_1_ retained its functional properties. Flow cytometry was used to study the ability of MNT_1_ to interact with EGFR. For this, MNT_1_ was labeled with the fluorescent dye AF488. Flow cytometry experiments demonstrated that cell fluorescence significantly increases after 15 min of incubation of cells with MNT_1_-AF488 (Figure 1). To determine the contribution of nonspecific (EGFR-independent) interactions, AML-12 cells were incubated for 1 h with an excess of EGF. In this case, cell fluorescence decreased by approximately three times (Figure 1). Consequently, the affibody in MNT_1_ retains its ability to interact with EGFR-positive AML-12 cells specifically in an EGFR-dependent manner. Thus, using radiolabeled MNT_1_ we demonstrated EGFR-specific binding and internalization of labeled MNT_1_ into EGFR-expressing A431 cells (Figure 5).

To test the functionality of the endosomolytic module within the MNT_1_ molecule, we evaluated the MNT_1_ ability to form defects in the model lipid membrane at pH values characteristic of endosomes. MNT_1_-mediated membranolytic activity peaked at pH values around 5.5–6.0 (Figure 2), which corresponds to endosomal pHs. This indicates that the endosomolytic module within MNT_1_ retains its functional activity.

It turned out that MNT_1_ possesses two Keap1 binding sites; one is the anti-Keap1 monobody while the other one is located on the HMP carrier module, thus promoting MNT_1_ interaction with Keap1. To demonstrate that MNT_1_ can interact with Keap1 not only in solution, but also in the living cells, the Förster resonance energy transfer fluorophore lifetime-determined microscopy method (FLIM-FRET) was used [32,33]. To do this, AML12 cells were transiently transfected with Keap1 protein fused to the green fluorescent protein hrGFP. It acted as a FRET donor molecule. The fluorescent dye AF568 attached to MNT_1_ and MNT_0_, acted as a FRET acceptor. In the presence of FRET between the donor and acceptor, i.e., when macromolecules labeled with them form a complex, the fluorescence lifetime of the donor is noticeably reduced [32,33]. Typically, the average fluorescence lifetime is determined by describing the fluorescence decline with a single exponential dependence. However, in the presence of FRET, not only the average lifetime of the donor fluorescence decreases, but also the decrease in fluorescence begins to be poorly described by a single-exponential dependence, i.e., the standard deviation, χ^2^, of the observed fluorescence drop approximated with the single-exponential dependence increases. When AML-12 cells containing Keap1-hrGFP were incubated with MNT_1_-AF568, we observed both a decrease in the average hrGFP lifetime and a noticeable increase in χ^2^ in a number of cell regions (Figure 3). This indicates the presence of FRET between Keap1-hrGFP and MNT_1_-AF568 in these regions, and hence the interaction between Keap1 and MNT_1_. Moreover, already 15 min incubation of MNT_1_-AF568 with these cells is enough for the average lifetime of hrGFP to significantly decrease, indicating that a significant proportion of MNT_1_ entered the cells from the beginning of its interaction with Keap1 (Figure 3e). In contrast, incubation of the cells with MNT lacking anti-Keap1 monobody, MNT_0_-AF568, does not significantly reduce the fluorescence lifetime of Keap1-hrGFP (Figure 3e). If MNT_1_-AF568 is added to AML-12 cells in the presence of excess EGF, the average fluorescence lifetime of Keap1-hrGFP is also not significantly changed (Figure 3e). This suggests that MNT_1_ not only binds preferentially to EGFR, as we have shown previously, but is also internalized into cells via binding to these receptors rather than via nonspecific internalization.

If we describe the hrGFP lifetime by a two-exponential dependence, with characteristic lifetimes t_1_ and t_2_, then we can calculate the FRET efficiency, E, using the following formula [32,33]:

E = 1 − t_1_/t_2_ (1)

It is then possible to obtain histograms of the occurrence of E values in AML-12 cells transiently transformed with Keap1-hrGFP, to which MNT_1_-AF568 was not added (Figure S2a, black curve) and after the addition of MNT_1_-AF568 (Figure S2a, red curve). Presumably due to the presence of endogenous acceptors for hrGFP, a peak is also observed in the histogram of the control, where MNT_1_-AF568 was omitted, at E values less than 22 (Figure S2a, black curve). Therefore, only E values above 22 were used for calculations. The difference in FRET efficiency histograms for AML-12 cells incubated with MNT_1_-AF568 and the control allows us to highlight the contribution of real FRET efficiency due to the added MNT_1_-AF568 (Figure S2b). The distribution of real FRET efficiency is well described by a Gaussian curve (Figure S2b, red curve). The maximum of this curve is 59.4 ± 0.6%. It should be noted that, knowing the FRET efficiency, E, one can calculate the average distance between donor and acceptor molecules using the following formula:

(2) $$r=r_{0}\cdot \sqrt[{6}]{\frac{1}{E}-1},$$

where *r*_0_ is the Förster radius [32,33]. For the pair of fluorophores used, hrGFP and AlexaFluor568, *r*_0_ = 5.4 nm (https://www.fpbase.org/fret/ accessed on 26 October 2023). Thus, we obtain *r* = 5.07 ± 0.02 nm. The spread of this value can be estimated from the half-width of the peak presented in Figure S2b. Therefore, the average distance between the hrGFP donor and the AF488 acceptor in the MNT_1_-Keap1 complex is *r* = 5.1 ± 0.8 nm. This is smaller than the characteristic size of similar MNTs, which is 8.3–10.6 nm [31]. From the peak area in Figure S2b, the proportion of the cell region in which FRET is observed can be determined. It is 22.5 ± 0.8% and reflects the proportion of Keap1 molecules involved in the formation of a complex with MNT_1_.

In summary, in AML-12 cells transiently transformed with Keap1-hrGFP, MNT_1_ is able to interact with Keap1. In order to understand whether MNT_1_ is able to interact with native intracellular Keap1 cellular thermal shift assays (CETSA) were used [34,35,36,37,38]. Using this method, so-called protein melting curves are obtained, which depend on whether the protein forms a complex with other molecules or not. The first is a melting curve of Keap1 in its natural protein environment in an AML-12 cell in the absence of MNT_1_ (Figure 4, blue curve). Next, AML-12 cells are lysed, and 1 μM MNT_1_ is added to the lysate before CETSA is performed and a melting curve is obtained. This curve will correspond to the Keap1 complex with MNT_1_ (Figure 4, red curve). When MNT_1_ is added to AML12 cells for 15 min with subsequent washing from unbound MNT_1_, the melting curve of Keap1 obtained by the CETSA method is the same as that of Keap1 in complex with MNT_1_ (Figure 4, black curve). When AML12 cells are also incubated with control MNT_0_, then the melting curve of Keap1 corresponds to cells incubated without MNT_1_ (Figure 4, green curve). These data allow us to suggest that MNT_1_ interacts with Keap1 in cells, and the main contribution to this interaction is made by the presence of the anti-Keap1 monobody within MNT_1_. Using FLIM-FRET, it was shown that MNT_1_ enters the cell in an EGFR-dependent manner. In the other words, MNT_1_ is supposed to enter cells via EGFR-dependent endocytosis. At a low temperature of 4 °C, the rate of endocytosis should drop sharply. The melting curve of Keap1 obtained at 4 °C demonstrated the absence of interaction of MNT_1_ with Keap1 (Figure 4, wine curve). This indicates that MNT_1_ enters the cell via receptor-dependent endocytosis. In summary, the results obtained using CETSA suggest that MNT_1_ enters the cell by endocytosis and is able to exit endosomes into the cytosol and interact there with Keap1.

So, we have shown by two different methods that MNT_1_ is capable of interacting with Keap1 in cells. It is known that Keap1 is able to bind to the PGAM5 protein, which is located in the outer membrane of mitochondria [40]. Therefore, it can be supposed that a noticeable part of MNT_1_ that penetrates into the cytosol will also be located on the surface of mitochondria. To test this assumption, we used FLIM microscopy; for this, we labeled the MNT_1_ with the fluorescent dye AF488, and stained mitochondria with MitoTracker RED FM. Unlike conventional confocal microscopy, this method allows the fluorescence of AF488 and MitoTracker RED FM to be clearly separated, both from each other and from autofluorescence. The fact is that the fluorescence lifetimes for all of them are significantly different. In addition, the magnitude of the autofluorescent signal was significantly lower than the magnitude of the dye signals. Together with separation by lifetime, this makes it possible to eliminate almost completely the contribution of autofluorescence from the resulting images and unambiguously determine the location of MNT_1_-AF488 and MitoTracker RED FM. The orange color in the image indicates that there is colocalization of these dyes in the same places within the cell (Figure 6). Moreover, a significant linear correlation between the fluorescence intensity of these dyes is observed (Pearson’s correlation coefficient is 0.77 at *p* < 0.001) in the places where they are recorded together (Figure 7). The Spearman rank correlation coefficient, which indicates the relationship between the fluorescence intensities of both dyes, is 0.87. The data obtained allow us to assume that MNT_1_ colocalizes with mitochondria in A431 cancer cells.

If MNT_1_ is mainly located in the mitochondrial region, then the delivery of a cytotoxic agent that can damage the mitochondrial membrane should lead to the death of target cells, presumably via apoptosis. To test this assumption, we used A431 cancer cells, which are known for their high level of EGFR expression, and in which we had already demonstrated the mitochondrial localization of MNT_1_. The photosensitizer chlorin *e*_6_ was chosen as a cytotoxic agent. As a result, we observed an 8.6-fold significant increase in the photocytotoxic effect of the photosensitizer attached to MNT_1_, compared to the free photosensitizer (Figure 8). On the contrary, attachment of the photosensitizer to MNT_0_ does not significantly increase its photocytotoxicity.

Thus, in this work, we proposed and studied MNT_1_, which is capable of interacting with the Keap1 protein located on the mitochondria inside the cells. MNT_1_ ability to interact with Keap1 within living cells was established. Colocalization of this MNT_1_ with mitochondria and a significant increase in the photocytotoxic effect of photosensitizers attached to MNT_1_ were shown. The obtained MNT_1_ is expected to be tested in vivo, just as we did earlier for modular nanotransporters carrying photosensitizers and aimed at delivery to the nuclei of target cells [20]. In addition, it is intended to study possible effects caused by MNT_1_ binding to Keap1, such as possible activation of the Nrf2 system [41,42] and changes in autophagic degradation of mitochondria [43].

## 5. Conclusions

As a result of this work, modular nanotransporters, containing monobodies to the Keap1 protein, were developed. Their ability to bind to the EGFR on the surface of target cells, disrupt the integrity of the lipid membrane at pH values characteristic of endosomes, and bind with high affinity to the Keap1 protein in solution were demonstrated. Using cellular thermal shift assay and FLIM-FRET microscopy, it was shown that the proposed MNTs interacted with the Keap1 protein not only in solution but also in living cells. Using FLIM microscopy, a noticeable co-localization of these MNTs with mitochondria was demonstrated. It was shown that the photosensitizer chlorin *e*_6_ acquired an 8.8-fold higher photocytotoxicity when it was coupled to the MNT and thus delivered to mitochondria. We assume that the proposed modular nanotransporters delivering biologically active molecules to a selected intracellular protein can open a new way to increase therapeutic effectiveness of chemo- and radiotherapy of malignant tumors.

## Data Availability

Data are contained within the article and Supplementary Materials.

## Supplementary Materials

The following supporting information can be downloaded at: https://www.mdpi.com/article/10.3390/pharmaceutics15122687/s1, Figure S1: Thermophoretic curves for MNT_0_ (a), Affibody (b), DTox (c), DTox-HMP (d), and Keap1. Fluorescence before the start of thermophoresis was taken as 100%. Fluorescence values 20 s after the start of thermophoresis are shown. Figure S2: (a) Frequency of occurrence of FRET efficiency, E, of AML12 cells transiently transfected with Keap1-hrGFP that were incubated for 1 h with 500 nM MNT_1_-AF568. The black curve shows the histogram for the control, in which MNT was not added to AML12 cells. (b) Difference in FRET efficiency histograms for AML12 cells supplemented with MNT_1_-AF568 and control. The red curve shows the interpolation of the experimental data from the Gaussian curve. All histograms were obtained by averaging data over 10–14 cells.
