# Supplementary material for: Modular Nanotransporters Delivering Biologically Active Molecules to the Surface of Mitochondria
Source: Pharmaceutics. 2023 Nov 27;15(12):2687. doi: 10.3390/pharmaceutics15122687 (PMC10748074; doi:10.3390/pharmaceutics15122687)
Supplement: Supplementary file 1 [file pharmaceutics-15-02687-s001.zip › pharmaceutics-2720305-supplementary.pdf]

Supplementary Materials: Modular Nanotransporters Delivering Biologically Active Molecules to the Surface of Mitochondria

Yuri V. Khramtsov, Alexey V. Ulasov, Tatiana A. Slastnikova, Andrey A. Rosenkranz, Tatiana N. Lupanova, Georgii P. Georgiev and Alexander S. Sobolev

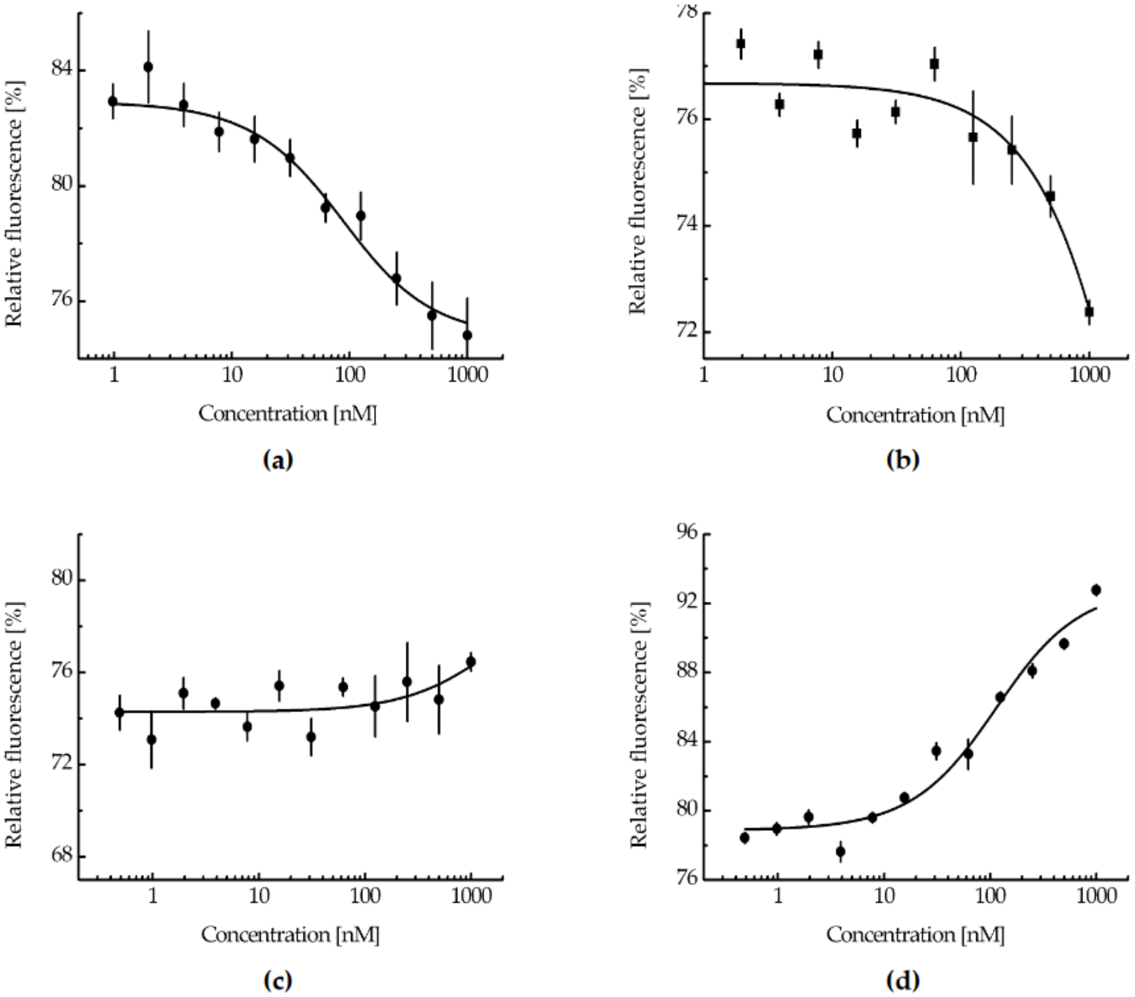

Figure S1. Thermophoretic curves for MNT_0 (**a**), Affibody (**b**), DTox (**c**) and DTox-HMP (**d**) and Keap1. Fluorescence before the start of thermophoresis was taken as 100%. Fluorescence values 20 seconds after the start of thermophoresis are shown.

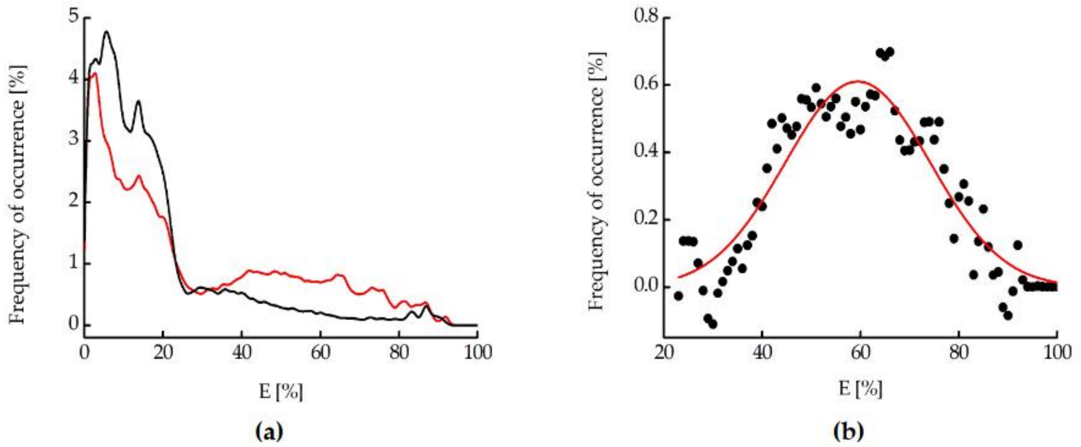

Figure S2. (**a**) Frequency of occurrence of FRET efficiency, E, of AML12 cells transiently transfected with Keap1-hrGFP that were incubated for 1 hour with 500 nM MNT_1-AF568. The black curve shows the histogram for the control, in which MNT was not added to AML12 cells. (**b**) Difference in FRET efficiency histograms for AML12 cells supplemented with MNT_1-AF568 and control. The red curve shows the interpolation of the experimental data from the Gaussian curve. All histograms were obtained by averaging data over 10-14 cells.
